# The diagnostic utility of thoracic ultrasonography in sheep and goats with contagious caprine pleuropneumonia

**DOI:** 10.3389/fvets.2025.1736810

**Published:** 2026-01-27

**Authors:** Mohamed Tharwat, Abdelmonem Abdallah

**Affiliations:** 1 Department of Clinical Sciences, College of Veterinary Medicine, Qassim University, Buraidah, Saudi Arabia; 2 Department of Health Management, Atlantic Veterinary College, University of Prince Edward Island, Charlottetown, PEI, Canada; 3 Centre for Veterinary Epidemiological Research, Atlantic Veterinary College, University of Prince Edward Island, Charlottetown, PEI, Canada

**Keywords:** caprine pleuropneumonia, diseases, diagnosis, mycoplasma infection, small ruminants, thoracic ultrasound

## Abstract

Contagious caprine pleuropneumonia (CCPP), caused by *Mycoplasma capricolum* subspecies *capripneumoniae* (*Mccp*), is a highly contagious respiratory disease that affects goats and, to a lesser extent, sheep. It remains a major cause of economic loss in smallholder farming systems, particularly in arid and semi-arid regions. Diagnosing CCPP in the field is challenging due to overlapping clinical signs with other respiratory diseases and limited access to confirmatory laboratory testing. Thoracic ultrasound (TUS) has emerged as a practical, non-invasive tool that enables real-time visualization of pleural effusion, lung consolidation, and fibrinous adhesions. Characteristic sonographic findings in affected goats and sheep include unilateral pleural effusion with echogenic fibrin strands, liver-like lung consolidation, and pleural septations. The utility of TUS extends beyond its established role in CCPP, offering a robust approach for the differential diagnosis of respiratory diseases in small ruminants. It facilitates timely and evidence-based clinical decision-making, supports the monitoring of therapeutic outcomes, and contributes to broader herd health management strategies. By bridging clinical and population-level applications, TUS demonstrates considerable potential as a frontline diagnostic modality to advance animal health, strengthen disease control programs, and promote sustainable rural livelihoods.

## 1 Introduction

Contagious caprine pleuropneumonia (CCPP) is a severe respiratory disease primarily affecting goats and, under certain epidemiological conditions, sheep. It is caused by *Mycoplasma capricolum* subspecies *capripneumoniae* (*Mccp*), a fastidious bacterial pathogen known for its high contagiousness (1). The disease manifests as an acute fibrinous pleuropneumonia, characterized by fever, cough, respiratory distress, and pleural effusions (2). Mortality rates during outbreaks can exceed 70%, causing significant losses to herds and considerable economic hardship for smallholder and pastoral communities reliant on small ruminant production (3).

CCPP is endemic in parts of Africa, the Middle East, and Asia, where goats and sheep are integral to rural livelihoods through meat, milk, and income generation. Outbreaks are often introduced via carrier animals and may result in morbidity and mortality rates approaching 90% and 60%, respectively (3, 4). Beyond animal health, CCPP imposes substantial economic burdens through reduced productivity, restricted trade, and increased veterinary costs (3).

Goats are the principal reservoir and most severely affected species, frequently experiencing acute clinical disease with high losses (1, 2, 5, 6). In contrast, sheep exhibit greater resistance, with natural infections being uncommon and typically subclinical, experimental infections resulting in only mild disease, and spillover in mixed flocks remaining inefficient and short-lived; consequently, sheep act primarily as incidental hosts (7–9). This interspecies difference reflects variation in host–pathogen interactions and immune responses, underscoring the apparent host specificity of *Mycoplasma capricolum* subsp. *capripneumoniae* (1).

Risk factors for CCPP outbreaks include close contact among infected goats and sheep, movement of infected animals, poor biosecurity, and environmental stresses such as overcrowding and harsh climatic conditions (10). Diagnosis is one of the most critical and challenging aspects of the disease, as it directly impacts the choice of prophylactic and therapeutic measures, as well as the strategies used to prevent its global spread (2). Definitive diagnosis relies on laboratory tests, including bacterial culture, polymerase chain reaction (PCR), and serological assays (11). An enhanced typing method based on Multi-Locus Sequence Analysis has been developed to improve resolution for tracing new epidemics and determining whether the recently identified cases in continental Asia resulted from the recent importation of *Mccp* (12). However, these tests are often impractical in field settings, leading to diagnostic delays that hinder timely intervention and allow rapid disease transmission (13).

Vaccination remains a key control strategy, but effectiveness is hampered by antigenic variability of *Mccp*, cold chain requirements, and logistical difficulties in vaccine delivery to remote areas (14). Moreover, lack of rapid and reliable field diagnostics results in frequent empirical antibiotic use, raising concerns about antimicrobial resistance (13). Recently, experimental studies have explored novel vaccine candidates targeting Mccp virulence-associated proteins, such as dihydrolipoamide dehydrogenase (DLD), which has been identified as an immunogenic adhesin involved in host cell attachment and plasminogen binding (15). These findings suggest that DLD may represent a promising antigen for future vaccine development; however, further *in vivo* studies are required to evaluate its protective efficacy, safety, and feasibility under field conditions.

Auscultation of the chest is a fundamental part of the clinical examination of sheep. Proper auscultation requires systematically examining all lung fields dorsal, ventral, and lateral on both hemithoraxes. Animals should ideally be standing or in sternal recumbency, and each pulmonary lobe should be assessed in defined intercostal regions to ensure comprehensive evaluation (16, 17). Nevertheless, routine interpretation of auscultated sounds often fails to detect superficial lung lesions or accurately determine their distribution in respiratory diseases.

Increased audibility of normal lung sounds can occur due to factors such as handling, transport, or endotoxaemia. In advanced pulmonary diseases, such as ovine pulmonary adenocarcinoma, coarse crackles may be heard over areas larger than the actual lesions. By contrast, focal pleural abscesses may produce no detectable abnormal sounds, while conditions like unilateral pyothorax or marked fibrinous pleurisy can cause attenuation of lung sounds, and pleural friction rubs are often not audible even in severe cases (18). Consequently, auscultation alone is insufficient for reliably diagnosing pulmonary pathology in small ruminants, highlighting the need for complementary diagnostic methods to improve lesion detection, localization, and severity assessment (17–19).

Thoracic ultrasound (TUS) has gained attention as a potential adjunct diagnostic method in sheep and goat medicine (20). This imaging technique allows real-time visualization of lung and pleural lesions such as consolidations, pleural effusions, and fibrin deposits without the need for sedation (21). TUS has been widely demonstrated to be effective in diagnosing various pulmonary conditions in adult small ruminants, including mycoplasmosis, viral infections such as ovine pulmonary adenocarcinoma (OPA) and lentiviral disease, parasitic infestations, and pseudotuberculosis (17, 22, 23).

In a previous case report (22), four ewes with interstitial pneumonia caused by Maedi–Visna virus (ovine lentivirus) were examined using TUS. Ventral lung abnormalities were identified, characterized by pleural thinning or effacement with confluent B-lines (“white lung”), loss of normal A-line artifacts, and parenchymal consolidation with a liver-like echotexture consistent with pulmonary hepatization. In a more recent study involving 111 lambs that evaluated the relationship between clinical scoring, auscultation, ultrasonography, and gross post-mortem findings in respiratory disease (17), TUS detected a range of abnormalities, including B-lines, pulmonary consolidation, pleural effusion, and abscess formation.

Current research in humans is exploring the role of TUS not only in diagnosing pleural diseases but also in predicting the outcomes of pleural interventions and personalizing treatment strategies based on ultrasound findings (24). While studies in both animals and humans have established the reliability of TUS for diagnosing respiratory conditions (25, 26), research specifically validating its use in goats and sheep affected by CCPP is still in the early stages. Although previous reviews have discussed the broader use of ultrasonography in diagnosing thoracic and abdominal infections in ruminants, they have generally adopted a multi-disease and multi-species perspective rather than focusing in depth on a single condition (21).

This review provides a disease- and species-specific overview of contagious caprine pleuropneumonia (CCPP) in sheep and goats. It integrates current knowledge on the bacteriology, immune response, epidemiology, clinical presentation, and control challenges of CCPP, while giving particular attention to the diagnostic role of TUS. We also aimed to highlight the differential diagnostic value of TUS in pulmonary pathologies of small ruminants and its potential integration into a structured workflow for diagnosing respiratory conditions in these species.

## 2 Epidemiology of CCPP

### 2.1 Transmission and risk factors

*Mycoplasma capricolum* subspecies *capripneumoniae* primarily infects the respiratory tract, with a strong affinity for pulmonary and pleural tissues, which contributes to the severity and localization of disease manifestations (27, 28). CCPP is highly contagious, spreading predominantly via aerosolized respiratory droplets during close contact between infected and susceptible goats and sheep (3). Contributing risk factors include overcrowding, stress (e.g., transport, sudden climate shifts), and poor ventilation (10). The disease is most common in arid and semi-arid regions, where nomadic practices and mixed herding of sheep and goats increase exposure (2). While *Mccp* does not survive long in dry environments, its persistence is prolonged under moist or cold conditions, and it can remain viable in frozen pleural exudate for years (3). Importantly, subclinical carriers play a key role in disease transmission, especially when they are moved into naïve herds or exposed to stressors (29).

### 2.2 Clinical signs and disease progression

**Figure 1:**
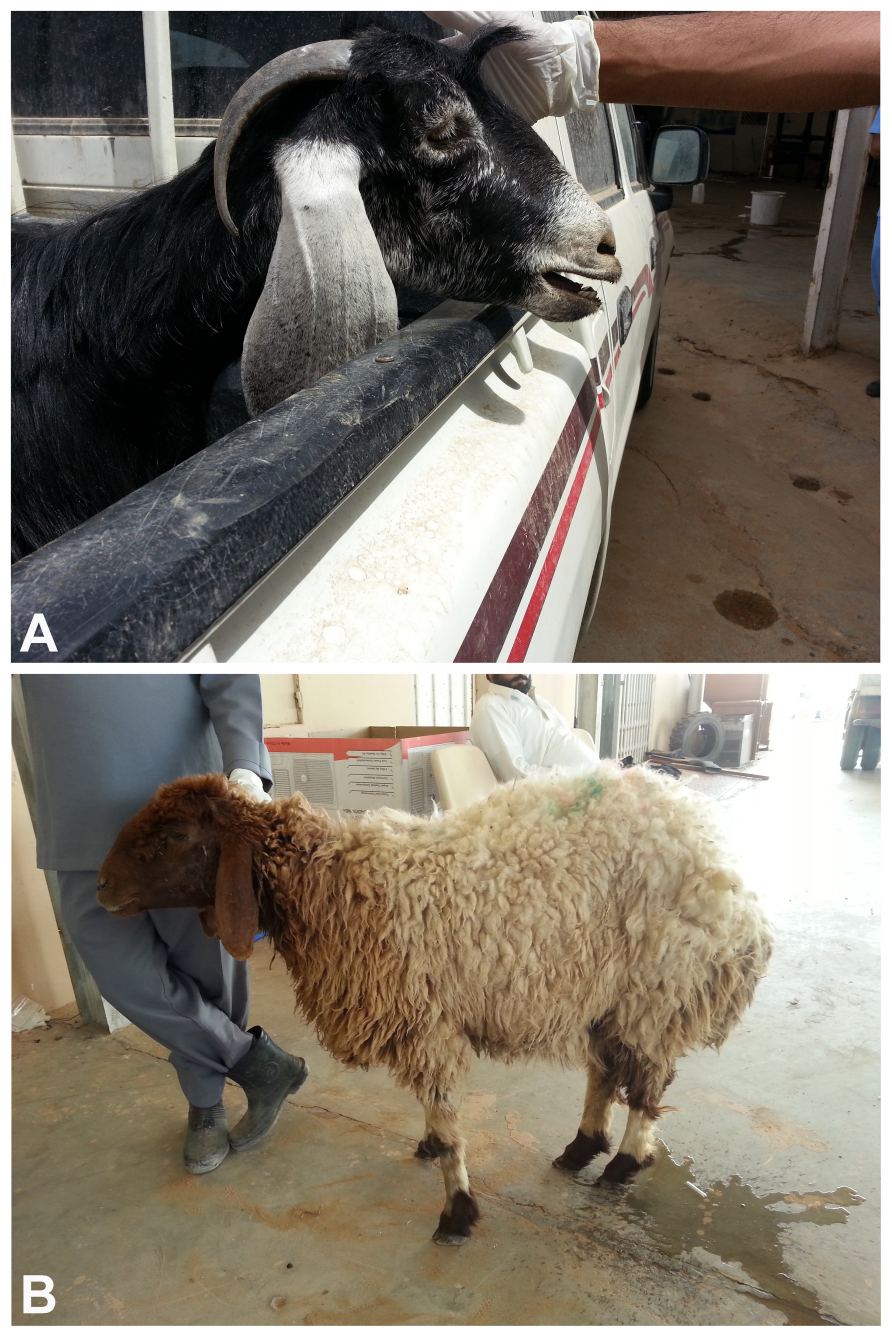
Clinical presentation of a goat **(A)** and a sheep **(B)** affected by contagious caprine pleuropneumonia. Observed clinical signs included anorexia, weight loss, fever, polypnea, dyspnea, coughing, tachycardia, cyanotic mucous membranes, nasal discharge, depression, and open-mouth breathing.

The clinical manifestation of CCPP can range from peracute to chronic forms. In peracute cases, sudden death may occur with minimal clinical signs (13). Acute presentations include high fever (41–43 °C), severe dyspnea, polypnea, nasal discharge, productive coughing, and pronounced postural adaptations such as extended neck and abducted forelimbs to ease breathing (3). In another study, the author reported that admission complains included weight loss, anorexia and respiratory signs that included nasal discharges polypnea, dyspnea, cough, metabolic acidosis, and open-mouth breathing in goats (30). In another recent report in sheep, authors found that clinical signs included fever, weight loss, polypnea, tachycardia, dyspnea, cough, nasal discharge, bluish mucous membranes, and open-mouth breathing (31) (Figure 1). Mortality in acute outbreaks may reach up to 60%−80%, with morbidity approaching 100% (1). Chronic cases often involve weight loss, intermittent coughing, and respiratory distress due to fibrous pleural adhesions and residual lung damage (2). Occasionally, diseased goats and sheep may die suddenly from complications such as pleural rupture or secondary infections (7). Pathologically, CCPP is characterized by unilateral fibrinous pleuropneumonia with massive pleural exudation, pulmonary consolidation, and adhesions between lung lobes and the parietal pleura (32). These changes create ideal conditions for sonographic detection (13).

### 2.3 Clinical and laboratory diagnosis of CCPP

Clinical assessment of CCPP relies on observing hallmark symptoms—high fever, respiratory distress, productive cough, and pleural pain evident through abdominal breathing and open-mouth posture (2, 13, 31). However, clinical diagnosis alone has limited specificity and sensitivity, as these signs overlap with other caprine respiratory diseases (3). Moreover, subclinical carriers present without obvious symptoms but remain infectious, further reducing diagnostic reliability (33). Isolation of *Mccp* from pleural effusion or lung tissues is considered the gold standard (34). Nevertheless, it is technically demanding, time-consuming, and requires specialized media and extended incubation periods—factors that limit its routine utility in most laboratories (2). Serologically, *Mccp* can be detected by complement fixation test (CFT), latex agglutination test (LAT), and competitive ELISA (cELISA) (35, 36). The LAT is reported to be more sensitive than the CFT and easier to perform than the cELISA (37). Molecular diagnostics include PCR-based assays that provide high specificity and sensitivity, especially in acute cases where bacterial load in pleural effusion is substantial (5). Multiplex and quantitative PCR approaches enhance pathogen differentiation and enable epidemiological tracking in small ruminants as previously reported (38).

### 2.4 Pathological anatomy of CCPP lesions

Postmortem examination of goats with CCPP reveals a range of gross lesions. The postmortem findings included pulmonary consolidation, pulmonary hepatization with a marbling appearance, turbid or serosanguineous pleural effusion containing fibrin, pleural abscessation, fibrinous pleuritis, and pleural adhesions, the pericardium may be thickened, opaque, and involved with hydropericardium and adhesions (2, 13, 32, 37, 39). In peracute cases, pulmonary edema may be the only finding, whereas chronic disease features progressive pleural adhesions and extensive lung hepatization (32). Similar but less severe lesions have been reported in sheep (5).

### 2.5 Ultrasonographic features

**Figure 2:**
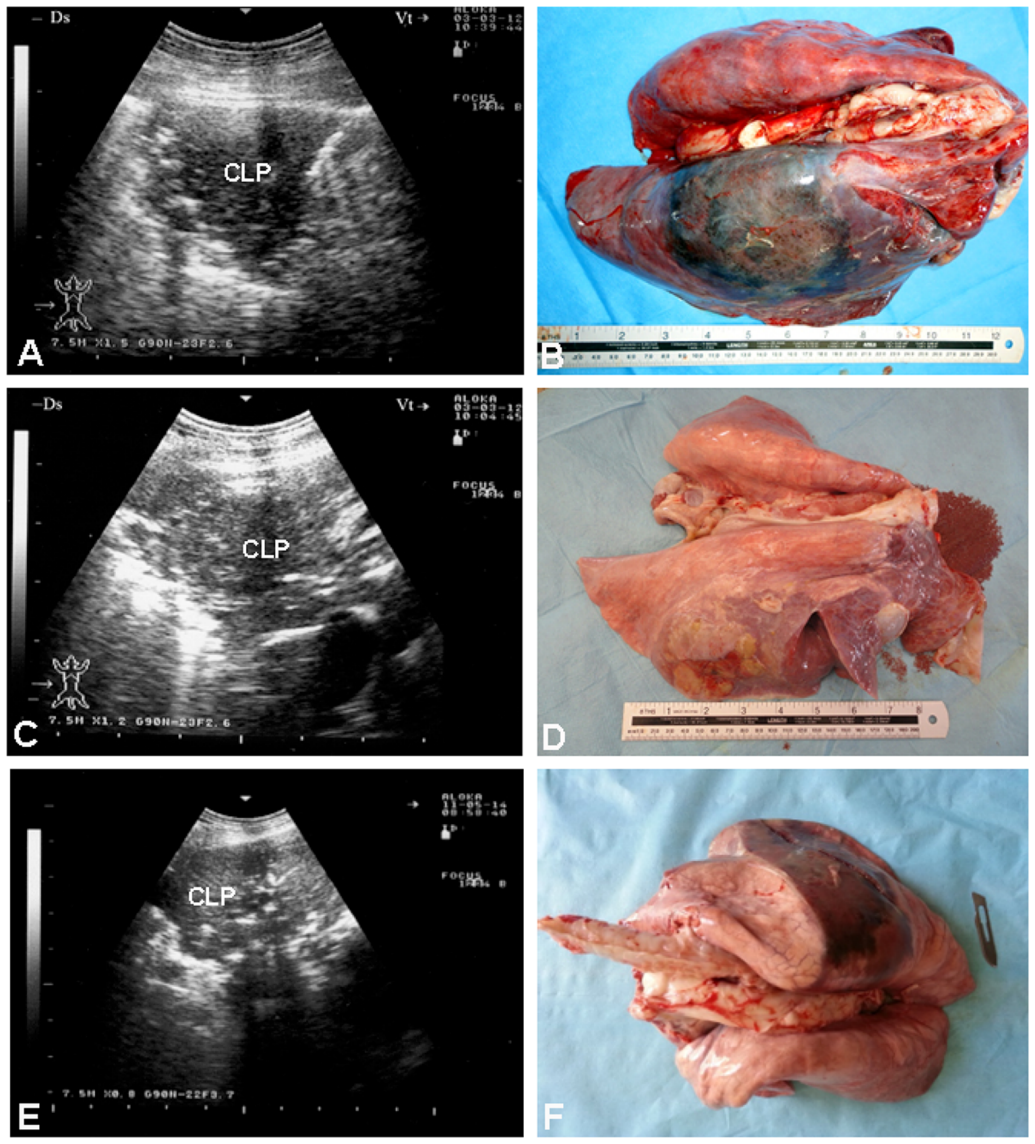
Gross and sonographic features of CCPP in goats and sheep. **(A, C)** show consolidated lung parenchyma (CLP) in two goats with CCPP, confirmed at postmortem examination **(B, D)**, respectively. **(E)** depicts a similar sonographic image in a sheep with CCPP, also confirmed at necropsy **(F)** [Reproduced from (13)].

Thoracic ultrasound excels at identifying hallmark lesions of CCPP—pleural effusion, pulmonary consolidation, and fibrinous exudates in large animals (18, 40). In small ruminants, ~24% of diseased goats show non-ventilated lung parenchyma with a liver-like echotexture on ultrasound (13). Depending on the extent of atelectasis, the underlying ventilated lung may be visible as faint, blurry, and well-defined reverberation artifacts (7). Post-mortem examinations confirm that the large hypoechoic areas in the cranioventral lung fields and the cranioventral regions of the main lobes correspond to consolidated lung tissue (41) (Figure 2).

**Figure 3:**
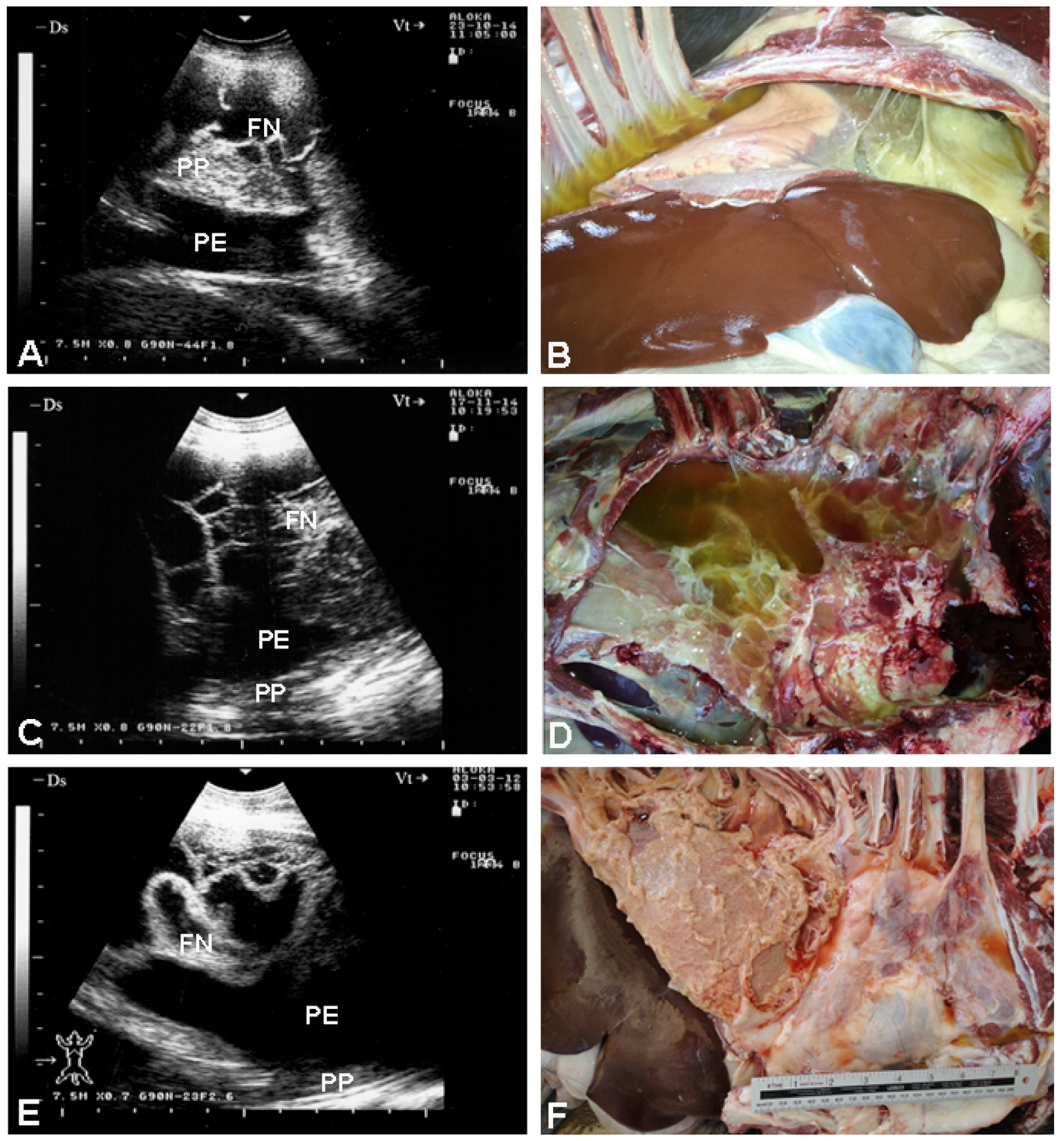
Ultrasonographic and corresponding postmortem findings in three goats with CCPP. **(A, C, and E)** reveal unilateral hypoechoic pleural effusion with echogenic fibrin strands (“tags”) and hepatized (liver-like) lung consolidation. Corresponding **(B, D, and F)** show postmortem confirmation, highlighting fibrin deposits that correlate with ultrasonographic echogenic tags—key indicators of disease severity and progression [*Reproduced from* (13)].

**Figure 4:**
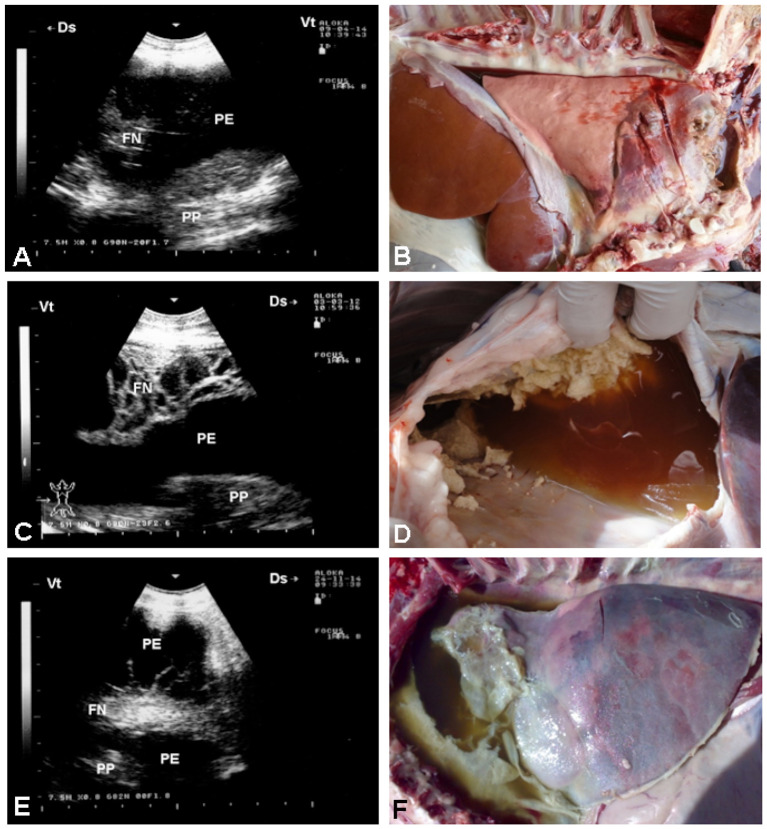
Ultrasonographic and postmortem findings in three sheep with CCPP. **(A, C, and E)** display hepatized lung parenchyma and pleural effusion ranging from anechoic to hyperechoic, with visible fibrin strands. Corresponding postmortem images **(B, D, and F)** confirm these sonographic findings. PE, pleural effusion; FN, fibrin network; PP, pulmonary parenchyma [*Reproduced from* (41), with permission by Open Veterinary Journal].

**Figure 5:**
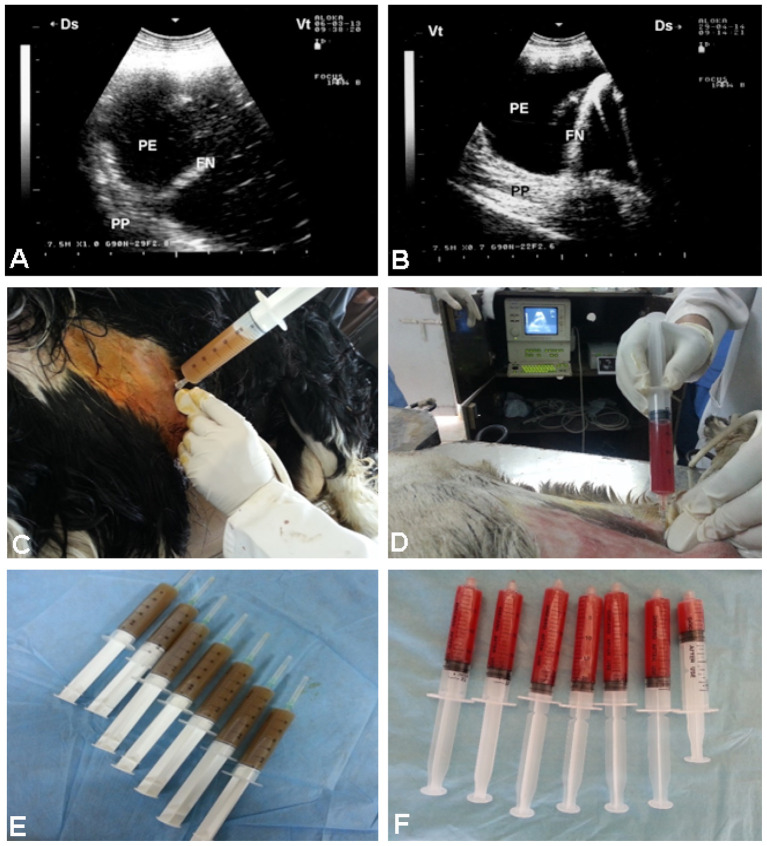
Ultrasound-guided pleural effusion aspiration in two sheep with CCPP. **(A)** shows hyperechoic pleural effusion (PE), a fibrin network (FN), and compressed pulmonary parenchyma (PP). Guided aspiration **(C)** yielded ~410 ml of turbid fluid **(E)**. **(B)** shows hypoechoic PE, FN, and compressed PP in the second case. Ultrasound-guided aspiration **(D)** collected ~127 ml of bloody fluid **(F)**. PE, pleural effusion; FN, fibrin network; PP, pulmonary parenchyma [*Reproduced from* (41), with permission by Open Veterinary Journal].

The vast majority of affected goats exhibit unilateral hypoechoic pleural effusions, often accompanied by echogenic fibrin strands or “tags,” and a “liver-like” appearance of consolidated lung parenchyma (7). Pleural adhesions appear as thickened septa separating fluid pockets. *In vivo* and post-mortem correlations confirm echogenic tags as fibrin deposits—critical markers of disease staging and severity (13). Always the postmortem investigation correlates well with antemortem sonographic findings (Figure 3). In sheep with CCPP, pulmonary ultrasound shows consolidated lung parenchyma with a liver-like texture. In addition, pleural effusion, the most prominent sonographic finding, exhibits echogenicity ranging from anechoic to hyperechoic, with fibrin networks and precipitates of varying severity. Necropsy findings correlate also well with the picture of sonography carried out antemortem (41) (Figure 4). Moreover, TUS enables longitudinal monitoring of CCPP and can improve treatment outcome (7). The presentation of dyspnea greatly improves following the procedure of ultrasound-guided aspiration of pleural effusion (41) (Figure 5). A comparative summary of the reviewed studies (Table 1) delineates the characteristic ultrasonographic findings for CCPP, their pathological confirmation, and the associated strengths and limitations of this diagnostic approach.

**Table 1:** Summary of study characteristics, ultrasonographic findings and diagnostic validation in goats and sheep with contagious caprine pleuropneumonia (CCPP).

**Study ID**	**Ultrasonographic features**	**Confirmatory diagnosis**	**Strengths**	**Limitations**
(13)–goats	Consolidated lungs appeared as liver-like echotexture Hypoechoic pleural effusion with thickened hyperechoic pleura Fibrinous pleurisy presented as a bright fibrinous matrix with anechoic pockets and displaced lung tissue Pyothorax showed hypo-/anechoic fluid with echogenic foci Pericardial effusion appeared hypoechoic with echogenic fibrin tags	Serological test: LAT for initial case selection Gold standard correlation: post-mortem examination confirmed all ultrasonographic findings	First study to detail ultrasonographic findings for CCPP caused by *Mccp* Post-mortem correlation for all ultrasound findings (gold standard validation) Provided clear diagnostic criteria for various lesions (e.g., consolidation, effusion) Highlighted a practical, non-invasive field tool for use where radiography is impossible Included a control group to define normal vs. pathological ultrasound appearance	Relied on the latex agglutination test alone for diagnosis, no culture or molecular confirmation Did not rule out co-infections with other pathogens via bacteriology
(41)–sheep	Consolidated lung parenchyma with a liver-like texture Pleural effusion (anechoic to hyperechoic) Fibrin networks (mild to massive) and precipitates within the pleural effusion Medial displacement and compression of lung tissue	Serological test: LAT for initial screening Post-mortem examination and histopathology used as gold standard confirmed the ultrasonographic findings	First study to document CCPP in sheep in Saudi Arabia Strong validation via correlation of ultrasound with post-mortem and histopathological findings Highlights a practical, field-friendly tool (ultrasound) for diagnosis and management (e.g., guiding aspiration to relieve dyspnea) Provides a comprehensive overview by integrating clinical, sonographic, and pathological data	Limited sensitivity in early stages: may not detect subtle initial changes Operator dependence: subjective interpretation can lead to inconsistent diagnoses Poor visualization of deep lesions: Challenging in obese animals or for dense, deep consolidations Limited specificity: findings like pleural effusion and consolidation are not unique to CCPP and can occur in other respiratory diseases

CCPP, contagious caprine pleuropneumonia; Mccp, Mycoplasma capricolum subsp. capripneumoniae; LAT, latex agglutination test.

While TUS offers a valuable, non-invasive tool for rapid, pen-side diagnosis of contagious caprine pleuropneumonia (CCPP), it should be regarded as one component of a comprehensive diagnostic and control strategy. Confirmatory diagnosis ideally requires integration of clinical assessment, ultrasonographic findings, and laboratory techniques described earlier. Nevertheless, TUS represents a practical on-farm tool for rapid case identification, particularly in remote settings where laboratory confirmation may be delayed or unavailable (30).

Evidence from dairy and beef production systems demonstrates that early detection of subclinical lung lesions is associated with improved clinical and production outcomes, underscoring the broader value of early respiratory disease diagnosis across livestock species (42–44). Calves presenting with lung consolidation at arrival are at increased risk of developing chronic pneumonia and exhibit reduced average daily gain, as well as lower carcass weight and quality (43). Early identification at purchase enables timely therapeutic intervention, potentially improving treatment success, and supports targeted management strategies, including specialized recovery diets, enhanced supportive care, and segregation of affected animals to limit disease transmission. Detection of pneumonia at purchase may also inform economic decisions, such as adjustment of purchase prices (45). In feedlot calves, TUS facilitates field-based assessments and supports informed decisions regarding antimicrobial therapy and herd management (46). Similarly, pulmonary ultrasonography has been reported as a convenient diagnostic technique for ovine respiratory complex in fattening lambs (17).

### 2.6 Management of CCPP

Control of CCPP in endemic areas requires a multipronged approach combining early detection, prompt isolation of affected goats and sheep, and appropriate antibiotic therapy to reduce bacterial shedding and disease progression (11). During severe outbreaks, strategic culling or slaughter of chronically infected goats and sheep may be required to control the spread of the disease. Vaccination, particularly using inactivated *Mccp* strains, has shown efficacy in reducing morbidity and mortality, although availability and strain coverage remain challenges (14). Improvements in husbandry—such as reducing animal density, ensuring adequate ventilation, and minimizing stress—also play critical roles in disease prevention and overall herd health (47). Thus, combining imaging modalities like TUS with these broader management strategies can enhance the early identification and control of CCPP outbreaks.

## 3 Application of thoracic ultrasound in pulmonary pathologies of small ruminants

### 3.1 Basic principles of thoracic ultrasonography

Thoracic ultrasound is grounded in the propagation and reflection of high-frequency sound waves at tissue interfaces (48). In healthy lungs, the aerated alveolar spaces act as a barrier, creating a bright pleural line with reverberation “A-lines” beneath it—artifacts produced by reflection from air–tissue interfaces (49). Lung sliding—seen as a shimmering motion synchronized with respiration—indicates normal pleural apposition, while diseased lungs with reduced aeration permit greater ultrasound penetration (50). Consolidation appears as tissue-like hypoechoic regions, sometimes with vertical artifacts as comet-tail “B-lines” or air bronchograms (51). A-lines usually appear in healthy lungs as horizontal, hyperechoic lines resulting from blockage of ultrasound wave propagation by the air contained within lung tissue (19, 52). However, in affected lungs, comet-tail artifacts (B-lines) appear as vertical lines that may be numerous, indicating localized pleural thickening or surface irregularity (45). Similar pathological artifacts have been reported in dairy buffaloes and cattle calves suffering from pulmonary emphysema and interstitial syndrome (53).

Pleural effusions show anechoic/hypoechoic fluid with floating debris or septa (7). This visual clarity enables detection of even small fluid volumes (13). Pleural thickening/adhesions manifest as irregular, non-sliding pleural lines, often indicating fibrinous changes (31).

### 3.2 Required equipment, portability, and practical scanning protocol

Thoracic ultrasound for small ruminants requires a portable ultrasound machine equipped with linear (7.5–10 MHz) or micro-convex probes (5–10 MHz), offering optimal resolution for superficial thoracic structures (21). Micro-convex probes are generally preferred for TUS in small ruminants due to their smaller footprint, wider field of view, and superior penetration depth (7). These features allow easier intercostal access and better visualization of pleural effusion and deeper lung lesions. While linear probes offer higher resolution for superficial structures, their limited depth and larger size make them less practical for comprehensive lung assessment in goats and sheep (7).

In addition, power source options include portable generators or rechargeable batteries, enabling field deployment may be required. Additional accessories may be required such as coupling gel, protective covers, and optional Doppler capability, useful for evaluating vascularization of pleural or mediastinal lesions (49). Linear ultrasound probes offer superior pleural surface detail, while micro-convex probes facilitate access between ribs, especially in smaller animals (54). Goats and sheep can be examined standing, in lateral, or sternal recumbency (55), and in sheep with dense wool, clipping may be necessary to improve probe–skin contact—a less critical issue in goats (56).

Systematic scanning of predefined intercostal sites allows ultrasonographic findings to be summarized into a standardized scoring system. The ultrasound staging system recently used to describe the ovine respiratory complex in lambs (17) categorizes lung lesions based on predefined ultrasonographic patterns, including A-lines, B-lines, consolidation (CON), pleural effusion (PE), and abscesses (ABS). Findings across all scanned sites are then summed to assign a severity score, reflecting the extent of pulmonary involvement: Score 0 indicates normal lungs with predominantly A-lines; Score 1, more than five sites displaying B-lines without consolidation; Score 2, more than five sites displaying B-lines with fewer than five sites showing consolidation; and Score 3, more than five sites with consolidation and/or the presence of pleural effusion or abscesses. This system provides an objective and reproducible framework for quantifying lung lesions and linking ultrasonographic findings to clinical severity.

### 3.3 Thoracic ultrasound and clinical assessment of pulmonary diseases in small ruminants

Thoracic ultrasound offers distinctive imaging patterns that support the diagnosis of pulmonary pathologies in small ruminants. Pleural effusion may be present only as attenuated lung sounds and appears ultrasonographically as anechoic fluid layers that deepen ventrally (56, 57). Fibrinous pleurisy, often clinically silent, is characterized by reduced sounds on the affected side and a lattice-like sonographic pattern with interspersed fluid (16, 57). In pyothorax, overlapping rumen sounds may obscure auscultation, while ultrasound reveals fluid with hyperechoic gas echoes (14, 57).

Pleural abscesses may go undetected when focal but, when diffuse, cause systemic signs such as fever, wasting and dyspnea; ultrasonography shows encapsulated hypoechoic to anechoic cavities (58–60). Pleuropneumonia is more clinically evident, with abnormal lung sounds, coughing, and pyrexia, while imaging demonstrates consolidated parenchyma with hyperechoic foci and pleural effusion (19, 56, 60). Further clinical and ultrasonographic details are summarized in Table 2.

**Table 2:** Comparative overview of clinical and ultrasonographic features of pleuropulmonary diseases in sheep and goats.

**Pulmonary pathologies**	**Clinical features/auscultation**	**Ultrasonographic features**	**References**
Pleural effusion	May not be detected by the farmer on routine inspection. Attenuated lung sound	Appear as anechoic area, increasing in depth as the probe moved ventrally	(56, 57)
Fibrinous pleurisy	Typically unnoticed; detected on flock screening. Reduced sounds on affected side, increased sounds on the normal one	A bright lattice-like pattern with interspersed anechoic fluid pockets over the chest wall, associated with medial displacement and compression of lung tissue, whose surfaces appeared as broad hyperechoic lines	(16, 57)
Pyothorax (empyema)	Reduced lung sounds on the affected side, increased on the normal side; rumen sounds may overlap lung auscultation	Anechoic areas with many hyperechoic dots caused by gas echoes bordered by a broad hyperechoic line	(56, 57)
Pleural abscess	Auscultation could not detect focal pleural abscesses up to 10 cm, with no clinical signs described. Wasting, dyspnea, fever, and cough were characteristic of diffuse or larger abscesses	Hypoechoic to anechoic areas with multiple hyperechoic spots and a broad hyperechoic abscess capsule due to acoustic enhancement, with loss of the pleural echo and absent reverberation	(18, 58, 59)
Pleuropneumonia	Abnormal lung sounds like wheezing, rubbing vesicular and murmuring sounds. Anorexia, pyrexia, coughing, dyspnoea and nasal discharge	Hypoechoic consolidated lung parenchyma containing hyperechoic foci representing fibrin, residual bronchi, or gas. Pleural effusion appears as an anechoic to hypoechoic space between the lung and chest wall	(19, 56, 60)

### 3.4 Overview of the sonographic diagnostic workflow for small ruminants' respiratory conditions

**Figure 6:**
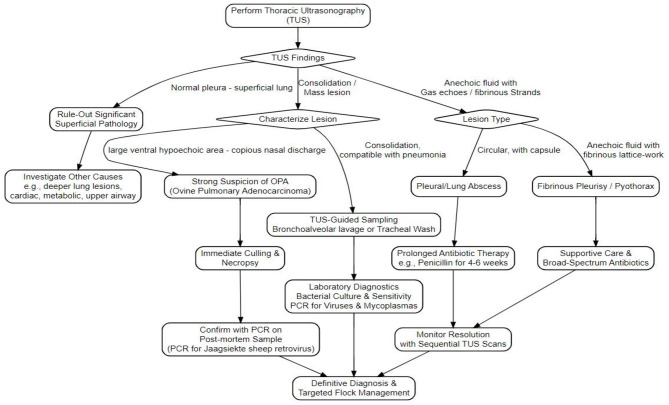
Stepwise diagnostic workflow to small ruminants' respiratory conditions using thoracic ultrasound (TUS). This figure was produced using information adapted from (16, 18, 38, 56, 57, 61).

To facilitate the clinical evaluation of respiratory disorders in small ruminants, a structured diagnostic workflow employing TUS has been established (Figure 6). This workflow consolidates characteristic ultrasonographic features to support differential diagnosis of prevalent pleuropulmonary conditions. This diagnostic pathway is based on established scientific literature, incorporating the research and clinical observations of experts in the field (16, 18, 38, 56, 57, 61).

The flowchart operates as a decision tree, starting with the initial ultrasonographic examination of the chest. It guides the user through a series of key questions based on the observed images, such as the presence or absence of the “gliding sign” (indicating normal lung movement), the appearance of comet-tail artifacts, and the characteristics of any abnormal fluid or tissue. Based on the answers, the workflow branches out to suggest possible diagnoses, which correlate with the pathologies detailed in Table 1, such as pleural effusion, fibrinous pleurisy, pyothorax, lung consolidation, and abscesses. The ultimate goal of the chart is to lead the practitioner from a generic clinical suspicion to a more specific sonographic diagnosis. Accordingly, when TUS identifies abnormalities such as pulmonary consolidation, pleural effusion, or mass-like lesions, additional diagnostics (e.g., targeted PCR, bacterial culture, or necropsy) should be undertaken promptly to optimize diagnostic accuracy and clinical decision-making.

This structured approach directly enhances diagnostic efficiency at the point-of-care, enabling quicker and more accurate differentiation between common pleuropulmonary pathologies such as pleural effusion, pyothorax, and consolidated pneumonia. Consequently, it empowers clinicians to move beyond presumptive diagnoses and make informed, evidence-based decisions. The ultimate benefit is the facilitation of earlier, more targeted therapeutic interventions and improved herd health management, solidifying TUS as an indispensable non-invasive diagnostic modality in the field.

### 3.5 Practical considerations for TUS

#### 3.5.1 Training, operator dependence, and cost-benefit analysis

One of the major constraints of TUS lies in its operator dependence. The accuracy of ultrasonographic interpretation is significantly influenced by the clinician's experience, anatomical knowledge, and familiarity with thoracic pathology (7). Variability in probe orientation, image acquisition, and misinterpretation of normal artifacts can lead to false positives or negatives, particularly in early-stage disease (62). As a result, a steep learning curve is associated with TUS, necessitating structured training programs and standardized protocols to improve inter-operator consistency (63). In recent years, portable ultrasound devices suitable for human and veterinary fieldwork have become increasingly accessible, with entry-level, battery-powered models (64) ranging from approximately USD 1,500 to 5,000, depending on brand and functionality. In contrast, radiographic systems often cost USD 10,000 or more, in addition to expenses associated with lead shielding, radiation safety compliance, and stationary infrastructure requirements (65). For rural and mobile veterinary services, the reduced logistical and safety demands of ultrasonography translate to a significantly lower operational cost profile over time (66). To reduce operator variability, standardized scanning protocols (defined intercostal spaces and quadrants), image acquisition settings, and training modules are essential. Tele-ultrasound mentorship and verified image libraries can help novices achieve diagnostic performance comparable to experienced operators (57). Thus, veterinary literature supports that with structured protocols, even convex and linear probes without shaving can produce reliable results in goats (57).

#### 3.5.2 Limitations

While TUS is a powerful tool, it has recognized limitations. Obesity or thick fleece particularly in sheep can impair sound wave penetration, reducing image quality and diagnostic yield (30, 56). In cases of severe pleural fibrosis or chronic disease, dense adhesions can obscure deeper structures or mimic neoplastic lesions, making interpretation more challenging (62). Pulmonary lesions not in contact with the pleura remain undetectable, as ultrasound cannot traverse air-filled alveoli. Thus, early parenchymal lesions or centrally located infections may be missed (63). Finally, in advanced disease stages, excessive fluid or consolidated tissue may limit anatomical differentiation, leading to under- or overestimation of disease extent (7).

## 4 Prospects and innovations

### 4.1 Knowledge gaps

Despite the growing application of thoracic ultrasound (TUS) in the diagnosis and management of CCPP, several important knowledge gaps remain. While TUS has proven effective in identifying pleural effusion, pulmonary consolidation, and fibrinous adhesions, there is currently no standardized, disease-specific TUS scoring system for CCPP that links ultrasonographic findings with disease severity, prognosis, or herd-level outcomes. Interpretation of ultrasonographic findings therefore remains largely descriptive and operator dependent, contributing to variability in diagnostic conclusions across clinicians and field settings.

In addition, the diagnostic performance of TUS is constrained by several technical and biological limitations. Sensitivity may be reduced during early stages of CCPP, as subtle parenchymal changes and mild clinical signs may not be detectable. Visualization of deep or densely consolidated pulmonary lesions can also be challenging, particularly in obese or large sheep, further limiting diagnostic accuracy. Moreover, key ultrasonographic findings such as pleural effusion and pulmonary consolidation are not disease specific and may occur in other respiratory conditions, complicating differentiation of CCPP from clinically similar disorders (41).

### 4.2 Future research directions

Artificial intelligence promises to enhance both the accuracy and consistency of TUS (64). AI driven tools employing convolutional neural networks have already improved detection of pleural effusion and pulmonary patterns in animal radiographs (64, 65), with cross-modal potential for TUS in ruminants. Veterinary imaging experts recognize AI not as a replacement but as a powerful decision-support tool—reducing operator variability, accelerating image interpretation, and flagging subtle lesions (66). Adaptation of these systems to TUS in small ruminants could facilitate instant lesion detection and scoring, improving diagnostic consistency in field environments (7).

To maximize the benefits of TUS, targeted and scalable training initiatives are essential. A notable example saw veterinary students mastering equine TUS in just 3 weeks using remote self-driven modules and handheld devices (67). Such frameworks could be adapted for small ruminants, combining online tutorials, hands-on workshops, and remote mentorship. In regions where paraprofessionals carry out herd surveillance, structured training programs—employing tele-ultrasound platforms—could dramatically improve early detection rates for CCPP. Field-deployable TUS systems, augmented with AI and telemedicine, can support rapid detection of zoonotic respiratory pathogens—protecting both livestock and communities (68, 69). Additionally, centralized databases of anonymized lung ultrasound images from sheep and goats could support epidemiological tracking of respiratory outbreaks, environmental risk factor mapping, and cross-species disease surveillance (7).

Emerging research (53) underscores TUS's role in not only identifying various pulmonary pathologies and assessing disease severity but also in monitoring treatment responses over time. Looking ahead, integrating TUS into routine veterinary practice could revolutionize the management of respiratory disorders in ruminants, enabling more precise tracking of disease progression and optimization of treatment protocols.

## 5 Conclusions

Thoracic ultrasound is emerging as a valuable, non-invasive diagnostic tool for managing CCPP and a wider spectrum of respiratory diseases in small ruminants. Its ability to distinguish between pleural effusion, pulmonary consolidation, interstitial involvement, and fibrinous adhesions provides significant advantages over clinical examination alone, particularly in differentiating CCPP from conditions like pasteurellosis or parasitic pneumonia. TUS also enables ultrasound-guided thoracocentesis, facilitating accurate sample collection for PCR or culture, which enhances diagnostic specificity and supports rational antimicrobial use. Beyond diagnosis, its role in monitoring treatment response and disease progression offers clear clinical management benefits. As portable ultrasound units become more affordable and training resources more accessible, TUS is increasingly feasible for use in field conditions, even in low-resource settings. Incorporating TUS into structured diagnostic workflows and national surveillance programs could substantially improve early detection, outbreak control, and herd-level decision-making. With further investment in training, standardization, and digital technologies such as AI-assisted interpretation, TUS has the potential to become a cornerstone of respiratory disease management in small ruminants—advancing animal health, productivity, and One Health goals across endemic regions.
